# A staging system for correct phenotype interpretation of mouse embryos harvested on embryonic day 14 (E14.5)

**DOI:** 10.1111/joa.12590

**Published:** 2017-02-09

**Authors:** Stefan H. Geyer, Lukas Reissig, Julia Rose, Robert Wilson, Fabrice Prin, Dorota Szumska, Ramiro Ramirez‐Solis, Catherine Tudor, Jacqui White, Timothy J. Mohun, Wolfgang J. Weninger

**Affiliations:** ^1^ Centre for Anatomy and Cell Biology & MICMedical University of ViennaViennaAustria; ^2^ The Francis Crick Institute Mill Hill LaboratoryLondonUK; ^3^ Wellcome Trust Centre for Human GeneticsOxfordUK; ^4^ Wellcome Trust Sanger InstituteHinxtonCambridgeUK

**Keywords:** Deciphering the Mechanisms of Developmental Disorders, embryo, high‐resolution episcopic microscopy, knock out, morphology, mutant

## Abstract

We present a simple and quick system for accurately scoring the developmental progress of mouse embryos harvested on embryonic day 14 (E14.5). Based solely on the external appearance of the maturing forelimb, we provide a convenient way to distinguish six developmental sub‐stages. Using a variety of objective morphometric data obtained from the commonly used C57BL/6N mouse strain, we show that these stages correlate precisely with the growth of the entire embryo and its organs. Applying the new staging system to phenotype analyses of E14.5 embryos of 58 embryonic lethal null mutant lines from the DMDD research programme (https://dmdd.org.uk) and its pilot, we show that homozygous mutant embryos are frequently delayed in development. To demonstrate the importance of our staging system for correct phenotype interpretation, we describe stage‐specific changes of the palate, heart and gut, and provide examples in which correct diagnosis of malformations relies on correct staging.

## Introduction

A number of research programmes coordinated by the International Mouse Phenotyping Consortium (IMPC) (Brown et al. 2006; Mohun et al. 2013) are currently exploring mammalian gene function through systematic phenotyping of mouse lines in which individual genes have been inactivated. In about one‐third of such lines, homozygous null individuals die prenatally (Ayadi et al. 2012; Hrabe de Angelis et al. 2015). In such cases, analysis of the morphological phenotype prior to embryonic death can offer important insight into genetic control of embryo development and also sheds light on the aetiology of congenital abnormalities (Mohun et al. 2013). Systematic study of embryonic lethal null mutations is underway in several centres, through programs such as ‘Deciphering the Mechanisms of Developmental Disorders’ (DMDD; https://dmdd.org.uk) (Mohun et al. 2013) and work of the Toronto Center for Phenogenomics (http://www.phenogenomics.ca).

These programs identify structural abnormalities in embryonic lethal null mutants harvested at several developmental stages, by comparison of mutant embryos with genetically normal counterparts. At the heart of all projects is morphological phenotyping of embryos that have just finished organogenesis, which is at E14.5–E15.5 (Weninger et al. 2014). Phenotyping during this window of development enables comprehensive analysis of the role gene products play in organ formation even when the defects resulting from gene mutation ultimately cause death during subsequent fetal stages. In the DMDD program, phenotyping occurs at E14.5, the earliest point when organogenesis is largely complete. This enables analysis not only of all embryos that have survived to this stage of development, but also allows examination of those that have died during the final stages of organogenesis. In such cases, despite the onset of autolysis, the DMDD program has found that significant useful phenotype information can still be obtained.

Comparison of digital volumes, produced by either micro‐computed tomography (μCT) (Wong et al. 2012) or high‐resolution episcopic microscopy (HREM) (Weninger et al. 2014), underpins efforts to identify the precise structural abnormalities affecting each embryo. However, whether such comparisons are performed by skilled morphologists (Mohun et al. 2013) or using automated software tools (Wong et al. 2012), their effectiveness is compromised by variations in developmental stage between embryos. Systematic analysis of wildtype embryos in the DMDD program has graphically illustrated the variation in size, morphology, topology and architecture of organs of embryos harvested at E14.5 from different litters, or even the same dam. Such variations profoundly complicate the identification of abnormalities, easily leading to erroneous interpretation of phenotype features and thereby resulting in false diagnoses of phenotypes. Without steps to address this problem, the reliability and usefulness of both individual embryo comparisons and systematic embryo phenotyping programs will be seriously compromised.

The essential first step is to define the range of normal morphology of embryos and their tissues as development proceeds, thereby establishing the variability that may be expected. A system for accurately classifying E14.5 embryos according to their developmental progress would help ensure that only embryos of equivalent developmental stage are compared and help minimise misdiagnosis of phenotypes. Furthermore, such a system would help identify developmental delays and heterochronic development of organs that may result from individual genetic mutations.

A potential classification system is somite counting, a commonly used approach for staging early embryos. However, by E14.5, accurate somite counting from external appearance is not feasible on account of the large number of somites and their differentiation. The system of staging described by Theiler (1989) provides a commonly used alternative. This is based on the appearance of several external features of the embryo, which change more or less dramatically during intra‐uterine development. E14.5 is considered to cover two Theiler stages (TS), TS22 and TS23 (Kaufman, 1992). Applying this system for phenotyping in the DMDD program has proved problematic for two reasons. First, division of embryos into only two stages (TS22 and TS23) proves to be insufficient for the speed and extent of internal morphological developments that occur during this developmental period. In addition, although Theiler staging uses several external features to establish a consensus classification of developmental stage, the same approach for staging at higher temporal resolution can be compromised by heterochrony between individual diagnostic features. Other staging systems (Wanek et al. 1989; Boehm et al. 2011), which are used in daily routine are not appropriate for distinguishing sub‐stages in E14.5 embryos (Table 1).

**Table 1:** Various staging systems for mouse embryos

	Böhm et al.	Wanek et al.	Theiler	Geyer et al.
Strain	C57BL/6 × CBA	Swiss‐Webster	C57BL/6 × CBA	C57BL/6N
Age	E10.5– E12.5	E10 ‐ PN5	E1–E19 (PN24)	E14.5
Stages	48	15	28	6
Fixed/unfixed	Fixed in 4% PFA	Unfixed	Unfixed/4% formol/Carnoy's solution/Bouin's solution	Fixed in Bouin's solution
Structure	Handplate	Handplate/footplate	Mutliple outer featuers	Handplate

To overcome this problem, we have used digital volume data from all the control embryos obtained in the DMDD program to develop a system for distinguishing six developmental stages in E14.5 embryos. Comparison of these has revealed a reliable time course for several normal developmental changes that occur during E14.5, each of which could be misinterpreted as a phenotypic abnormality with less precise staging. Applying this new staging for phenotyping mutant embryos in the DMDD program has therefore improved diagnostic accuracy and revealed the extent of developmental delay that frequently accompanies embryonic lethal gene mutation.

## Material and methods

### Embryos

We used 58 mouse lines of the C57BL/6N strain generated by the Wellcome Trust Sanger Institute (http://www.sanger.ac.uk/) as part of the DMDD project (https://dmdd.org.uk) and its pilot (Mohun et al. 2013; Weninger et al. 2014). From each line, 1–5 control embryos (total of 215) and 2–10 mutants (total of 297) were analysed (Table 2). All the mouse production and procedures were performed according to local ethical committee guidelines.

**Table 2:** Mouse lines used. Asterisk labels pre‐DMDD pilot study lines

1700007K13Rik	Chst11	Hdac1*	Psat1*
1700067K01Rik	Chtop	Hdac8*	Psph
2510003E04Rik*	Cir1	Ikbkb*	Pth1r
4933434E20Rik	Cmip	Jarid2*	Rhot1*
Akap9*	Cntfr*	Lmnb2*	Rundc1
Aldh18a1*	Col4a3bp	Ltbp1*	Sh3pxd2a
Amfr*	Csrp2bp*	Mbtd1*	Slc25a20
Anks6	D930028M14Rik	Mks1*	Slc5a7
Arid2*	Dbn1	Mybphl	Smpd4
Arid4a*	Dscc1*	Nf1*	Ssr2
Atp11a	Esco2*	Nxn	Supv3l1*
Brd2	Exoc3l2	Pds5b*	Tcf7 l2
Cbx1*	Fam134c*	Polb	Traf6
Celf4	Fam46c	Prkab1*	Zc3hc1*
Cenpj*	Gap43*		

### Generation of digital volume data

Embryos were harvested at E14.5 into Bouin's fixative for 24 h. They were then washed in phosphate‐buffered saline, dehydrated in methanol (10% steps until 90%, followed by 95% and 100%; at least 2 h each) and embedded in resin (JB‐4, Polysciences) containing eosin B and acridine orange, as previously described (Weninger et al. 2006; Mohun & Weninger, 2012a). Within each block, the embryo was oriented to secure transverse sectioning from crown to rump. Resin blocks were allowed to polymerise overnight at room temperature, baked at 90 °C for 24–48 h and then subjected to digital volume data generation using the high‐resolution episcopic microscopy (HREM) (Mohun & Weninger, 2012b). HREM data was downsized as appropriate to provide a standard voxel size of 3 × 3 × 3 μm^3^.

### Data analysis

Software packages amira 5.4 and osirix v5.6 were employed for data analysis. 3D volume models (threshold level 200 – 50) were used for staging from external morphology. A recently published protocol was used for phenotyping (Weninger et al. 2014).

### Biometric data

In volume‐rendered 3D models, the crown‐rump length (Fig. 4A) and the distance between the ventral tip of the lower jaw and the anterior edge of the ostium of the external auditory meatus (Fig. 4B) were measured. A software‐generated stack of sagittal sections was used to identify and measure the largest proximo‐distal extension of the tibia, analogous to the determination of fetal femur length in human obstetric ultrasound (Fig. 4C).

Statistics were performed using the software packages excel (Microsoft Excel for Mac 2011, Version 14.5.4) and spss (IBM SPSS Statistics for Mac Version 20).

## Results

### Developmental stage of DMDD embryos

**Figure 1:**
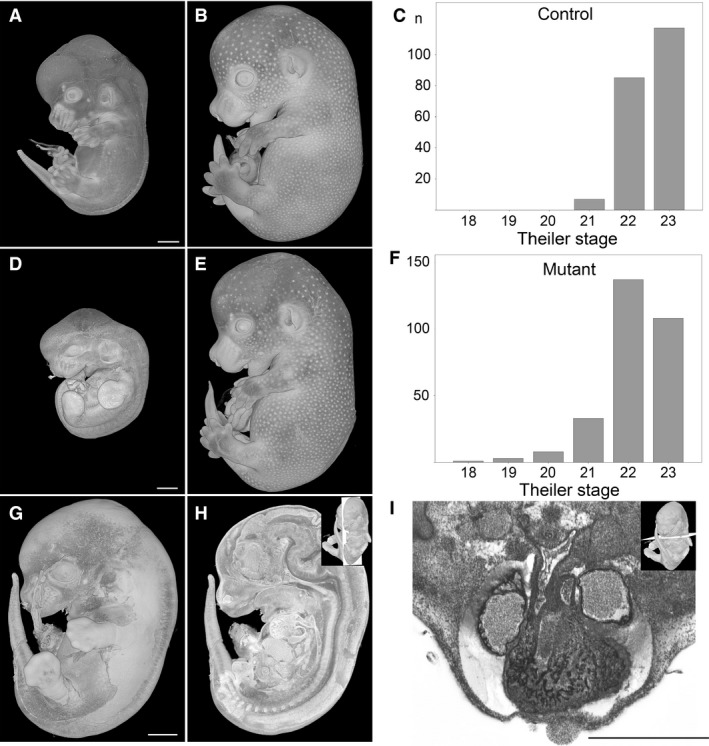
Developmental progress of E14.5 embryos. (A–C) Theiler stages (TS) of control embryos range from TS21 (A) to TS23 (B). (D–F) TS of mutants range from TS18 to TS23 (E). Note that panel D shows a TS19 mutant. (G–I) TS21 mutant in which autolysis has started. Scoring of the major organs and detection of malformations such as double outlet right ventricle (I) is possible. (A,B,D,E,G) Volume‐rendered 3D models. (H) Volume‐rendered model sectioned in the median sagittal plane. (I) Axial HREM section. Scale bars: 1 mm.

The developmental progress of the DMDD embryos harvested at E14.5 varied considerably, whether wildtype or mutant, irrespective of whether embryos were littermates. Profound differences were evident in surface morphology between the youngest and oldest control as well as knock‐out embryos (Fig. 1A,B,D,E). One of the lines produced only mutants that were already dead during harvesting and the tissues had started to be become autolytic (Fig. 1G–I). Despite this, comprehensive scoring of most of the features, including almost all components of the cardiovascular system in such embryos, was still feasible. In another five lines, mutants proved to be a mixture of autolytic and still alive at the time of harvest.

To classify the developmental progress of all E14.5 embryos, we first scored the developmental stage using the criteria proposed by Theiler. Using this system, control embryos were classified as Theiler stages TS21, TS22 and TS23, with 98% belonging in the group TS22/TS23. Mutants were scored as belonging to TS18–TS23, with 85% in the group TS22/TS23 (Fig. 1C,F).

### Alternative staging and sub‐stages

**Figure 2:**
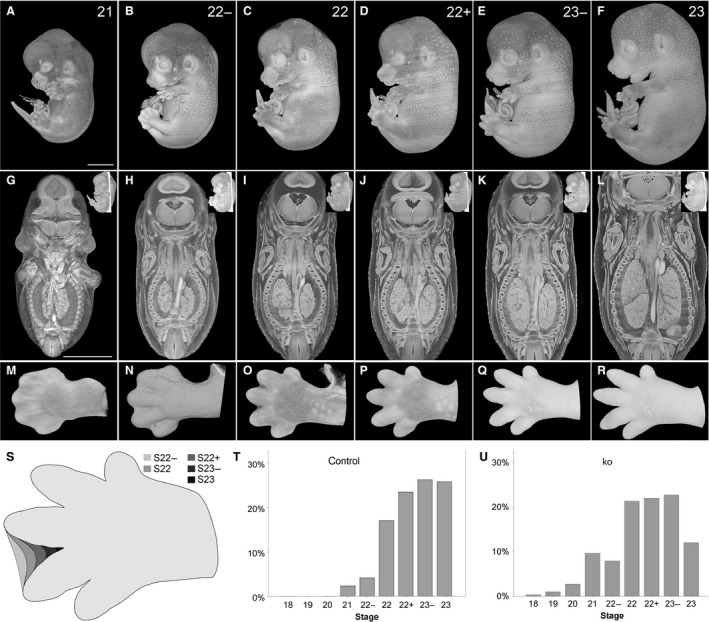
Stages of E14.5 embryos. **(**A–F) Volume‐rendered 3D models of the surface showing stages (S) 21–23. (G–L) Appearance of internal structures in coronal sections through the 3D models. (M–R) Volume‐rendered 3D model of hands of embryos classified as S21–S23. All models are set in their original, stage‐dependent size relations. (S) Scheme of development of interdigital space between 3rd and 4th digit from S22− to S23. (T,U) Stages distribution of DMDD control (*n *=* *215) (T) and knock‐out embryos (*n *=* *297) (U). Scale bars: 2 mm.

While scoring the organs of the embryos classified as TS22 and TS23, it became apparent that the appearance of the internal structures also varied considerably between the Theiler stages (Fig. 2G–L). We therefore examined which feature proposed by Theiler might provide a means of sub‐dividing TS22 and TS23 into sub‐stages. In the period TS21–TS23, the forelimb gradually and predictably changes its shape, starting as a paddle at TS21 and becoming a hand with separate fingers by TS23 (Fig. 2M–R). We therefore evaluated whether forelimb morphology alone could provide a reproducible, simple and accurate way to assess embryo stage solely on the basis of quick external observation. All 215 wildtype embryos were staged in the order they were produced and imaged, first according to Theiler criteria and then independently using the appearance of the forelimb. Both scorings yielded predominantly the same results, assigning 91% of the embryos to the same stage.

We then tested whether forelimb morphology permits distinction of developmental sub‐stages using the extent of interdigital webbing between digits 3 and 4 (Fig. 2S). Embryos showing a paddle with dorsal and palmar indentations between the forming fingers were classified as stage (S) 21. Embryos where the border of the interdigital web reached up to ¼, ½, ¾, and more than ¾ proximally to a virtual line running in the middle between digits 3 and 4 were staged as S22−, S22, S22+ and S23−, respectively. Embryos where the interdigital web had completely disappeared were staged as 23. Using this approach, we were able to distinguish six stages among the 215 control embryos harvested at E14.5: S21, S22−, S22, S22+, S23−, S23 (Fig. 2T,U).

### Correlation of staging system with biometric data

**Figure 3:**
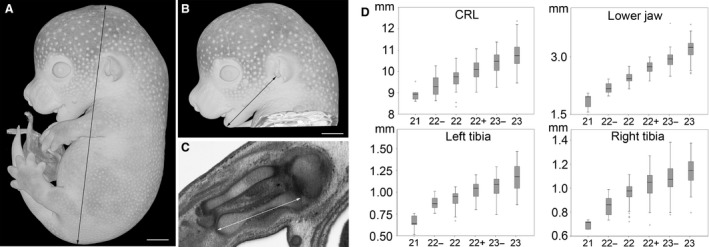
Biometry. Crown‐rump length (A) and dimension of lower jaw (B) were measured on volume‐rendered 3D models. Tibia lengths were measured in best fitting orthogonal HREM‐sections (C). The measurements are presented as box plots (D). Scale bars: 1 mm.

Having established that the forelimb provided a convenient feature to define developmental sub‐stages in E14.5 embryos, we assessed its reliability by comparing forelimb appearance with data gained from objective metric measures of embryo crown/rump length, length of lower jaw, and length of tibia (Fig. 3). Comparison with crown‐rump length (CRL) showed a Spearman's correlation coefficient of 0.624 (*P *<* *0.001), with the length of lower jaw (mandible) the value was 0.831 (*P *<* *0.001). Spearman's correlation coefficients of 0.675 (*P *<* *0.001) and 0.628 (*P *<* *0.001) were found with length of left and right tibia, respectively. As an additional non‐metric, but regardless of objective parameter, we also assessed the presence of hair follicles on head and body and again found a correlation of 0.729. Together, these results indicate that forelimb appearance reliably reflects embryo development as judged by multiple independent criteria in the S21–S23 range.

### Developmental delay of mutants

We next categorised all DMDD mutants according to the new staging system. Their external appearance was examined and the limbs were checked for obvious defects such as polydactyly or syndactyly. If the limbs appeared to be free of such defects, they were used for staging. We then compared the distribution of embryos among developmental sub‐stages between mutant and control embryos. A Wilcoxon Mann–Whitney *U‐*test revealed a significant difference between the two populations, with the mutants being significantly younger than controls (*P *<* *0.001). To investigate this further, we monitored the developmental progress of embryos harvested from each of the examined lines separately (*n *=* *58). In 21 lines (36%) at least one mutant embryo was younger than S22−. As control embryos are older or equal to S22−, we define embryos harvested at E14.5 but developmentally earlier than S22− as delayed in their development.

Among the many phenotypes identified in recessive lethal null embryos (https://dmdd.org.uk), those resulting in restricted oxygen and nutrient supply to the growing tissues might be expected to result in developmental delay. Indeed, 31% of mutant embryos identified with severe cardiovascular defects were younger than S22− and were thus considered delayed in their development.

### Impact of staging on phenotyping screen

**Figure 4:**
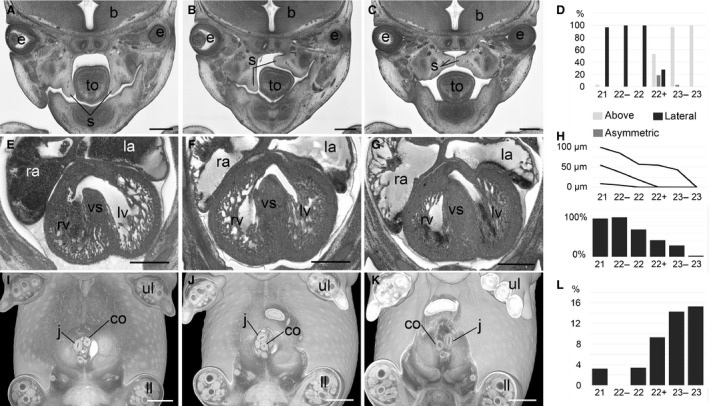
Examples for stage dependency of important anatomical features. (A–D) Position of palatine shelves (s) in coronal sections (A–C) and bar graph (D). (E–H) Appearance of interventricular foramen in axial sections (E–G), graph of percentiles (5%, 95%) and median of size of interventricular foramen in μm (above) and bar graph (H). (I–L) Rotation of intestine in volume‐rendered 3D models (I–K) and bar graph (L). b, brain; co, colon; e, eye; j, jejunum; la, left atrial appendix; ll, lower limb; lv, left ventricle, ra, right atrial appendix; rv, right ventricle; to, tongue; ul, upper limb; vs, ventricle septum. Scale bars: 500 μm.

Embryo organs and internal structures can show dramatic, stage‐specific differences during E14.5 and these can only be resolved using developmental staging that distinguishes sub‐stages within TS22 and TS23. This becomes obvious in virtual coronal sections through three‐dimensional (3D) volume‐rendered models (Fig. 2G–L). As a result, accurate scoring of phenotypes depends on an appreciation of stage‐specific changes in the anatomy and topology of the organs in embryo structures. Three important examples for which diagnosis is challenging in TS22 and TS23 embryos are:

*Cleft palate*. Cleft palate (MP: 0000111) is an important abnormality. It hinders sucking in the neonatal period and consequently may be responsible for perinatal death. The cleft is the result of improper closure of the left and right palatine shelves, which initially develop lateral to the tongue, shifting upwards to fuse in the midline. Almost all DMDD mutants, including those at TS23, show cleft palate. However, examination of control embryos reveals a surprisingly broad variety of positions for the palatine shelves. Reclassifying developmental stage using the forelimb handplate system, it is possible to define a developmental sequence that accounts for this apparent morphological variability. From S21 to S22, palatine plates are positioned laterally to the tongue. From S22+ they start elevating, but in an asymmetric manner. As a result, S22+ embryos can show one shelf above, and one shelf lateral to the tongue (Fig. 4A–C). Finally, from S23− onwards, all embryos have both shelves above their tongues, but only 35% have them yet fully fused along the midline. Having resolved this as a consistent normal developmental sequence, DMDD mutants are only diagnosed as showing cleft palate if they are older than S22+ and still do not have both shelves positioned above the tongue.

**Figure 5:**
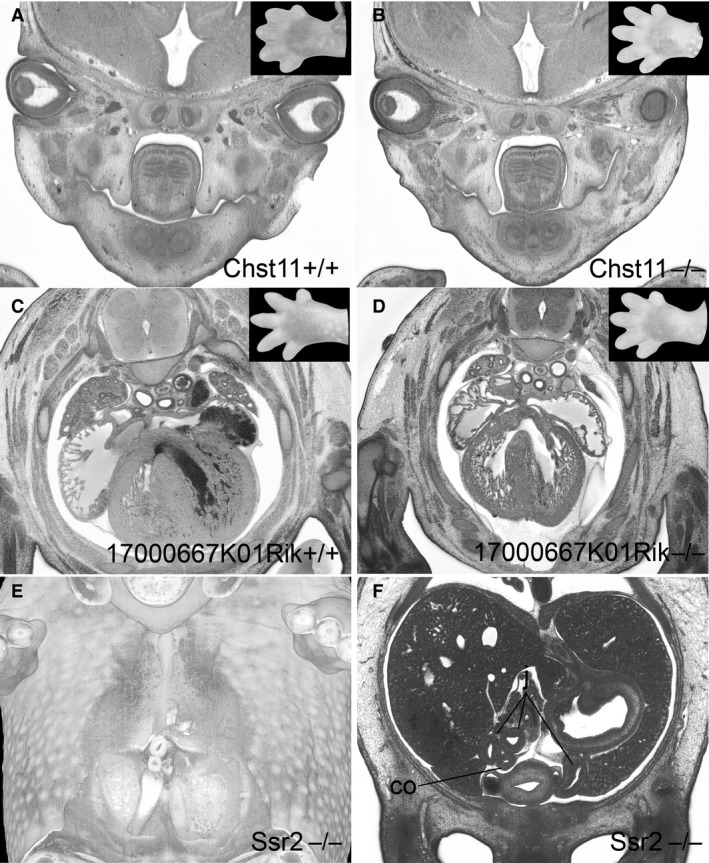
Impact of stage variations on phenotype scores. (A,B) Cleft palate in a control embryo (S22) and Chst11 mutant (S23−) (C,D) embryo (S23) and a 17000667K01Rik mutant (S22+) (C,D). The inlays show corresponding handplates. (E,F) Volume‐rendering (E) and original axial HREM‐section showing abnormal placement of the intestine in an Ssr2 mutant. co, colon; j, jejunum.

An example, of such diagnosis is the Chst11 knock‐out line, shown in Figure 5. Both the control (S22) and mutant (S23−) embryos appear to show cleft palate, but because of differences in their precise developmental stage, only the mutant can be scored with confidence as having palatine cleft (Fig. 5A,B).

*Ventricular septal defect*. Ventricular septal defects (MP: 010402) are severe heart defects which may occur in the muscular or membranous part of the septum. Both will cause severe haemodynamic problems. Postnatally, they will cause a mixing of oxygenated and non‐oxygenated blood in the ventricular chambers, resulting in reduced oxygen levels in the systemic circulation. However, in normal embryo development the ventricular septum grows from the apical region of the developing heart until it completes separation of left and right ventricular chambers by fusing at around E14.5 in the complex of tissues that form the atrio‐ventricular junction. An interventricular ‘foramen’ normally exists in developing embryos until the four‐chamber structure of the heart is complete.

Among E14.5 embryos analysed in the DMDD program, a gap in the interventricular septum has frequently been observed, varying widely in size and appearing to comprise a perimembraneous ventricular septal defect (pVSD). However, systematic examination revealed that 68% of control embryos also showed apparent pVSDs of varying size. When these were sorted by developmental sub‐stage, it became clear that both the size of the gap in the interventricular septum and the percentage of embryos showing it, decrease gradually from S21 to S23 (Fig. 4E–G). This therefore constitutes the developmental period during which septum formation is completed. Any minor gap is therefore most likely to be a remnant of the closing interventricular foramen rather than a pVSD. Distinguishing between these two is essential for accurate phenotyping and requires both an assessment of the size of the gap and the precise developmental stage of the embryo.

As an example, in the 17000667K01Rik line a mutant (S22+) showed a gap in the ventricle septum. The control embryo (S23) did not. As the gap in the septum (55 μm) falls within the range detected in normal embryos at an equivalent stage, we score this as a remnant of interventricular foramen, rather than as a pVSD (Fig. 5C,D).

*Abnormal placement of intestine*. Abnormal placement of the intestine (MP: 0014023) is another abnormality that requires careful assessment of developmental stage. It can be detected inside the embryo body, inside the physiological umbilical hernia, or where the intestine leaves and re‐enters the body. Older DMDD mutants frequently showed unusual placement of the intestine, prompting us to examine the changes in intestine topology that occur during E14.5. In younger control embryos (S21–S22), the jejunum is located solely on the right‐hand side inside the body, entering the umbilical hernia from the right (Fig. 4I). In contrast, the caecum is located on the left, inside the umbilical hernia, and the colon re‐enters the body on the left to run caudally to the anus. In 9% of S22+ embryos and 15% of the S23− and S23 embryos, the intestine had commenced its physiological rotation, placing the jejunum slings on the left within the embryo body, the caecum on the right inside the umbilical hernia, and the jejunum first cranially and then left of the colon at the connection of the umbilical hernia to the body of the embryo (Fig. 4I–L). As a result of this complex rotation, intestinal placement can appear highly variable and apparently abnormal in individual embryos unless their precise developmental stage is established. In the DMDD program, only patterns that differ significantly from any of the topologies seen across S21–S23 are scored as abnormal placement of the intestine. Embryos younger than 22+ showing a rotated intestine are considered to show heterochronic rotation.

As an example, in an Ssr2 knock‐out embryo (S22), the intestine was already rotated and jejunum slings already protruded to the left inside the belly. As this precisely matches the topology normally seen at S23 after rotation of the intestine, this embryo was scored as showing ‘heterochronic rotation of the intestine’ rather than ‘abnormal placement of the intestine’ (Fig. 5E,F).

## Discussion

From accumulated studies of the many genetically altered mouse lines, it appears that discrete windows within embryo development are particularly sensitive to genetic mutations that result in embryonic lethality. A small proportion cause embryonic death very early in development, presumably as a result of targeting genes involved in fundamental early cell fate choices or in establishing adequate placental support. A second sensitive period is midway through gestation, mutations disrupting heart development rapidly compromising survival of the entire embryo. However, in approximately one third of the lines producing pre‐ or perinatally lethal offspring, embryos complete the major period of organogenesis and reach a point when organ arrangement largely resembles that of the adult (E14.5–15.5). Among these, early assessments suggested that at least 60% would exhibit structural abnormalities (Mouse Genome Database (MGD), http://www.informatics.jax.org), a prediction amply confirmed by subsequent systematic studies (Mohun et al. 2013). Programs studying embryonic lethal null mutations have for these reasons adopted morphological phenotyping with embryos harvested at E14.5–15.5 as their primary screening procedure (Adams et al. 2013; Mohun et al. 2013).

Here we have used data from the DMDD program to investigate the impact of lethal mutations on the rate of developmental progress prior to lethality. We have found that mutant and control embryos harvested at E14.5 are remarkably heterogeneous in their appearance, with homozygous null mutant embryos often developmentally younger than their wildtype littermates or control embryos harvested at an equivalent time *post coitum*. Without more accurate developmental staging, direct phenotype comparisons of mutants with controls, (even with those harvested from the same dam) are therefore prone to result in false diagnosis of developmental delay and in misinterpretation of size, topology and structure of organs. Important examples for such misinterpretations are presented in this paper and involve the diagnosis of serious malformations, such as developmental delay, cleft palate and interventricular septal defect.

We therefore suggest a simple system for classifying the developmental progress of E14.5 embryos on the basis of scoring the appearance of the handplate. Obviously, hand plate maturation, like organ maturation, is not an abrupt but continuous process, which we have artificially divided into six stages. Nevertheless, the proposed system provides a rapid, reliable and simple way to identify the relative developmental stage of embryos falling within Theiler S22 and S23 and does not require measuring devices or special equipment. It is applicable to all embryos except the small number in which limb development is specifically targeted by the mutation. Fortunately, malformations of the upper limb have proved to be extremely rare, with just one case detected until now within DMDD project. The system we propose can be used with direct observation of embryos or with virtual volume models derived from 3D imaging procedures. Its simple approach makes it useful for a wide range of phenotyping studies, irrespective of the imaging technique on which such studies are based.

Our study was driven by the need to establish an accurate system for staging volume‐rendered 3D models produced by HREM imaging from Bouin‐fixed and resin‐embedded embryos. It is well known that fixation and dehydration leads to shrinkage by up to 10% of all embryonic tissues (Kaufman, 1992). It can not be ruled out that the digits and interdigital webs might be affected to a slightly different extent, raising the possibility that staging of native and fixed embryos could give different classification of some embryos. It remains to be evaluated whether embryos should be considered slightly “older”, if staged using 3D models.

Visualising volume‐rendered 3D models requires the definition of threshold values for selecting the features that should be displayed. In volume data of low resolution and with low tissue contrast, the selection of slightly different thresholds will cause small features, such as the interdigital webs to appear in different sizes. However, our models are derived from HREM data of high resolution (3 × 3 × 3 μm^3^) and high tissue contrast. As a result, the range of threshold values used to display the embryo surface have little discernible impact on the appearance or size of the handplate and interdigital webs.

Commonly used staging systems (Table 1) are insufficiently precise to permit assessment of structures subjected to rapid remodelling at E14.5 (Theiler, 1989; Wanek et al. 1989). Using handplate morphology as we have described has allowed six stages to be distinguished and has ensured that the scoring of abnormalities in the DMDD program is based on the comparison of mutant embryos with appropriately staged controls. The importance of this is exemplified by the difficulties in diagnosing cleft palate, perimembranous ventricular septal defects and abnormal intestinal morphology we have highlighted. Other similarly error‐prone diagnoses may well exist, but their identification will require systematic analysis of embryonic features and the compilation of biometric data.

Our study demonstrates that the common practice of comparing mutants and normal littermates for diagnosing abnormalities is fundamentally compromised. Not only do littermates vary significantly in developmental stage but, in addition, mutants are more likely to be developmentally delayed compared with normal embryos. As both size and topology of organs and tissues can change markedly during the TS22–23 window, accurate identification of abnormalities is only possible with developmentally stage‐matched embryos.

Alternative methods for ensuring accurate staging and appropriate comparison of embryos have previously been described but each require much more complex computational approaches, which may limit their utility. One such procedure also uses the morphology of the handplate, but staging is based on mathematical characterisations of handplate curvature and focuses on the earlier developmental period of E10.5–12.5 (Boehm et al. 2011). Its extension and applicability to E14.5 is, as yet, uncertain. A more comprehensive approach has recently been described that relies on computational comparison of entire volume datasets to provide a sophisticated and user‐independent assessment of precise developmental stage (Wong et al. 2015). This is an attractive and powerful approach but requires both the acquisition of a developmental baseline from imaging a large number of whole embryos and specialist computational methods that are not widely available.

The C57BL/6 strain was the first mouse strain with a fully sequenced genome. Consequently, the C57BL strain has been used in 45% of all studies working with inbred mouse strains (224,775 citations in PubMed) and selected by the IMPC for its phenotyping. Since the DMDD project forms part of the overall embryo phenotyping programme coordinated by the IMPC, we developed our staging system for this strain. It remains to be established how useful it will be with other strains which may differ in their precise rate of developmental progression. Nevertheless, forelimb morphology is likely to provide accurate relative developmental staging of embryos sharing a similar genetic background.

Mutant embryos with malformed or broken forelimbs cannot be staged using the limb. Our results show that changes in other easily accessible features, such as the crown‐rump length, length of tibia, length of lower jaw, or even the presence of hair follicles can be used as alternatives because these features correlate well with handplate morphology. All of these alternatives have serious problems as alternatives to the handplate. The presence of hair follicles will not permit distinguishing six sub‐stages in E14.5 embryos; tibial and lower jaw length are much less convenient as a general method for staging, as they require whole volume datasets for accurate measurement and the length of the tibia increases not strictly symmetrically or linearly. Even the simpler measurement of accurate crown‐rump length can be challenging, as the proportional changes across E14.5 are relatively small. Furthermore, reliable measurement of crown‐rump length is not possible in embryos showing a range of phenotypes such as exencephaly, anencephaly, severe scoliosis, or caudal regression.

The DMDD program scores phenotypes at E14.5, but the twin observations of the importance of precise stage comparisons for phenotyping and the prevalence of developmental delay in mutant embryos will no doubt apply at other time points, such as E15.5. By choosing the earlier time point, DMDD is able to identify and examine mutant lines that are autolytic at E14.5. This is a significant proportion, accounting for around 12% of the lines studied. Many of these show a mixture of autolytic and live embryos at the time of harvest. It seems likely that this reflects the wider finding from our studies that most phenotypes are not fully penetrant. It seems likely that autolytic embryos comprise those more profoundly affected by the phenotypes resulting from the particular gene deletion. Even in such cases, HREM analysis enables a useful degree of morphological assessment for the main organ systems, despite the evident degeneration of tissue integrity.

We showed that correct interpretation and characterisation of the phenotypes of mutant embryos harvested at E14.5 relies on distinguishing sub‐stages, defined by the maturing forelimb. It is to be expected that other developmental stages, especially around the edge of the embryonic to the fetal period, might likewise profit from more precise staging and systems can be envisaged, which for example are based on other easily accessible features, such as the developing lower limb. However, which events and features best fit which stages remains to be researched.

One third of the embryos studied here, representing 32 of 58 lines, had severe defects of the cardiovascular system. The majority of these embryos were much younger than their control littermates. Many were younger than TS22 and a few even appeared as if harvested as early as E12.5. We assume that cardiovascular defects restrict oxygen and nutrient supply to the embryonic tissues, slowing the growth of the embryo and its organs. Under such conditions we might expect the developmental delay to affect other organs in the embryo similarly, whether they showed additional phenotypes or not. Finally, it is noteworthy that a small proportion of control embryos harvested at E14.5 were developmentally younger than S22−. It remains to be seen whether these simply comprise outliers in the normal distribution of developmental progress, or whether their retardation results from functional abnormalities not detectable by aberrant morphological phenotype.

## Competing interests

No competing interests declared.

## Author contributions

S. H. Geyer wrote the paper, prepared figures, collected and analysed data. L. Reissig, J. Rose and D. Szumska phenotyped embryos. R. Wilson processed and managed image data. F. Prin generated image data. R. Ramirez‐Solis, C. Tudor and J. White produced knock‐out lines, produced and harvested embryos. T. J. Mohun contributed to assembling the paper and designing the project. W. J. Weninger phenotyped embryos, contributed to assembling the paper and designing the project.

## Funding

This work was supported by funding from the Wellcome Trust (WT100160). The DMDD research programme is funded by the Wellcome Trust (WT100160). It is also supported by the Francis Crick Institute, which receives its core funding from Cancer Research UK (FC001157, FC001117), the UK Medical Research Council (FC001157, FC001117), and the Wellcome Trust (FC001157, FC001117). T.M. was supported by the Medical Research Council (U117562103).
